# Unveiling the complexities of lung transplantation in situs inversus

**DOI:** 10.1186/s40792-024-01812-1

**Published:** 2024-01-19

**Authors:** Chitaru Kurihara, Taisuke Kaiho, Ankit Bharat

**Affiliations:** 1 Division of Thoracic Surgery, Northwestern University Feinberg School of Medicine, 676 N. Saint Clair Street, Suite 650, Chicago, IL 60611 USA; 2 Division of Pulmonary and Critical Care Medicine, Northwestern University Feinberg School of Medicine, Chicago, IL USA

**Keywords:** Lung transplant, Situs inversus, Respiratory failure

## Abstract

**Background:**

Lung transplantation for situs inverse is considered technically challenging because of the reverse positioning of the organs. By providing a detailed description of the surgical procedure, perioperative care, and post-transplant follow-up, we aim to contribute valuable information to the existing knowledge base. We presented two cases of successful bilateral sequential lung transplantation in situs inverse patients.

**Case presentation:**

Our first patient was a 28-year-old, non-smoking woman with Kartagener syndrome and advanced bronchiectasis that developed into pneumonia and required repeated hospital admissions. She underwent double lung transplantation. During the lung transplant procedure, venoarterial extracorporeal membrane oxygenation (VA ECMO) support was provided. The recipient’s morphologically right (anatomically left) lung was explanted. The right main bronchus was anastomosed, followed by the pulmonary artery and left atrial anastomoses. Afterward, we proceeded with the left side. Similar to the right side, left pneumonectomy and implantation were performed using the same methods. The duration of VA ECMO support was 147 min with a 328-min ischemic time. Because of the significant size mismatch, nonanatomic lung volume reduction over the right middle and left upper lobes was necessary. The patient had no complications postoperatively and was discharged on post-operative day (POD) 12. Our second patient was a 51-year- old man with scleroderma-associated interstitial lung disease with situs inversus. Bilateral sequential lung transplantation was performed. Similar to case 1, a clamshell incision was made at the fourth intercostal space entry. The patient then received VA ECMO support identical to that in case 1. The total VA ECMO support time was 155 min with 295 min of ischemic time. The patient recovered uneventfully and was discharged on POD 13.

**Conclusions:**

Lung transplantation for situs inverse can be a viable treatment option without modifying established transplantation procedures.

## Background

Lung transplantation is a well-established and life-saving procedure for patients with end-stage lung disease. Situs inversus, a rare congenital condition characterized by the reverse positioning of organs, poses unique challenges for surgical interventions, including lung transplantation. The scarcity of reported cases involving lung transplantation in situs inversus reflects the limited understanding of this complex condition. And, literature on lung transplantation in situs inversus remains sparse. Existing reports have documented a handful of cases, each presenting its own set of challenges and outcomes. Despite the limited number of reported cases [1–6], there are significant gaps in our knowledge regarding lung transplantation in situs inversus. Addressing these gaps is crucial for optimizing patient outcomes and refining surgical techniques in this unique scenario. The uniqueness of these cases lies not only in its rarity but also in the opportunity to gain insight into surgical techniques for lung transplantation in situs inversus. By providing a detailed description of the surgical procedure, perioperative care, and post-transplant follow-up, we aim to contribute valuable information to the existing knowledge base.

## Case presentation

### Case 1

**Fig. 1:**
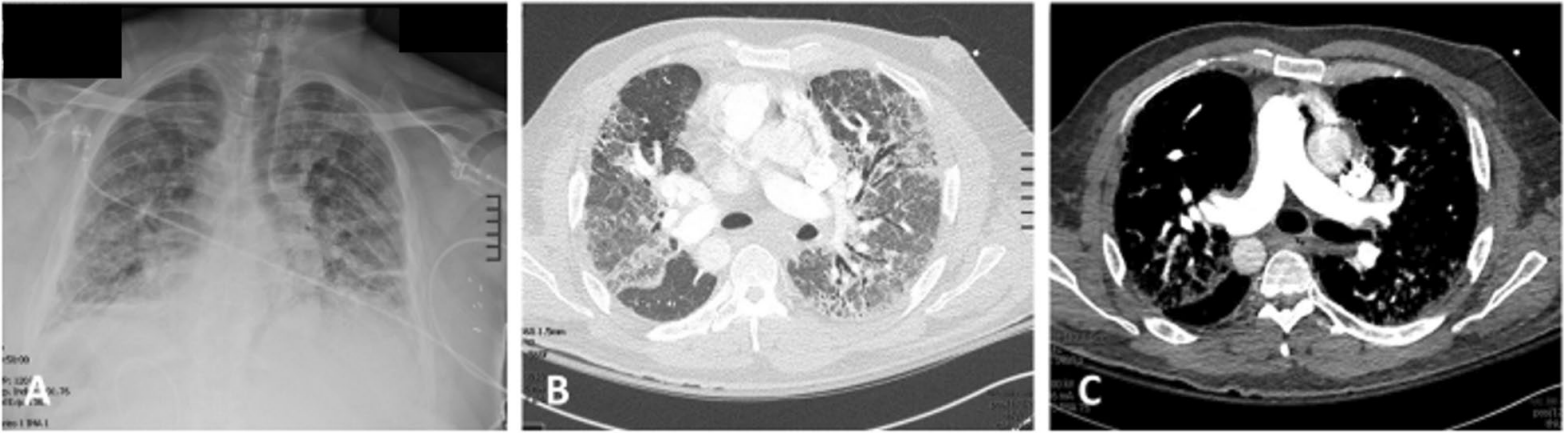
Chest X-ray and computed tomographic images (**A–C**), obtained prior to the lung transplantation, showing evidence of interstitial lung disease changes with situs inversus totalis

**Fig. 2:**
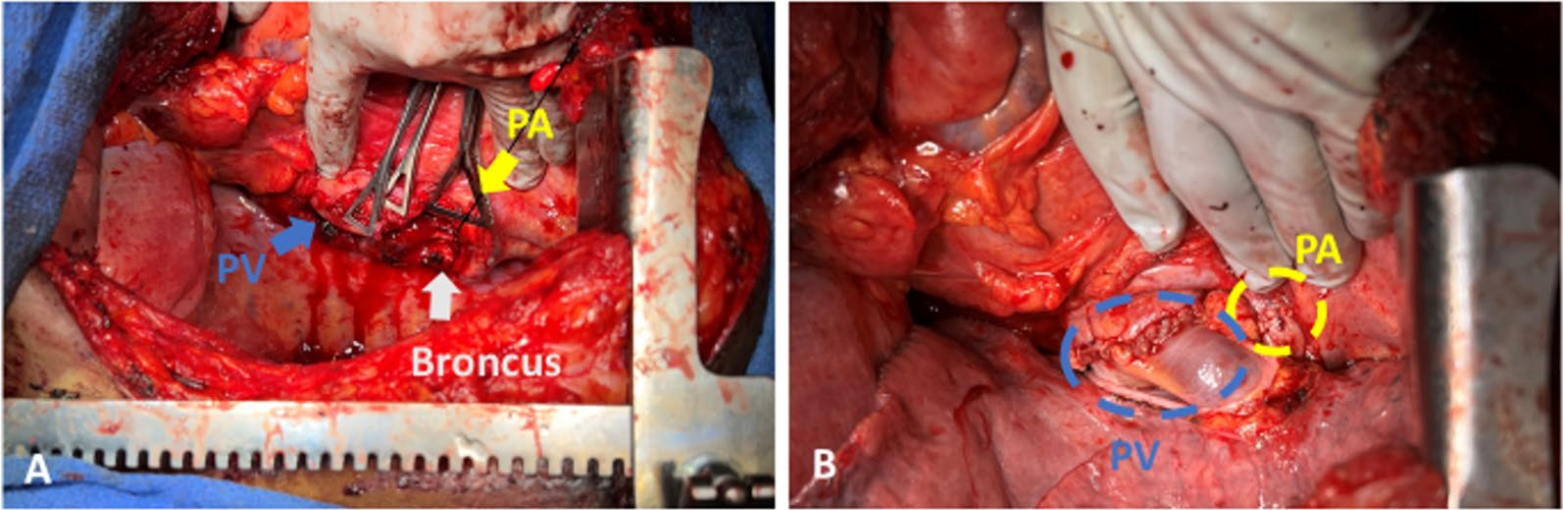
Intra-operative findings of pre- (**A**) and post-anastomosis (**B**) in left lung. *PA* pulmonary artery, *PV* pulmonary vein

Our first patient was 28-year old, non-smoking woman with Kartagener syndrome and advanced bronchiectasis that developed into pneumonia requiring repeated hospital admissions (Fig. 1). She underwent a bilateral, sequential lung transplant. During the lung transplant procedure, a clamshell incision was made at the fourth intercostal space entry. We then inserted a Medtronic EOPA 18F arterial cannula (Medtronic, Dublin, Ireland) and a triple-stage venous cannula (Medtronic, Dublin, Ireland) into the ascending aorta and right atrium, respectively. Venoarterial extracorporeal membrane oxygenation (VA ECMO) support was provided using a Quadrox-iD adult (7.0) oxygenator (MAQUET Holding B.V. & Co. KG, Germany) and rotaflow pump (MAQUET Holding B.V. & Co. KG, Germany). The recipient’s morphologically right (anatomically left) lung was explanted. The right main bronchus was anastomosed using 4-0 monofilament nonabsorbable suture and corner sutures were tied down so to avoid the purse-string effect. The PA was centrally clamped and then stapled were excised. The PA donor and recipient were anastomosed using 5-0 monofilament nonabsorbable suture. Last, PVs were clamped centrally and the vein stumps were excised. The bridge between the vein stumps were excised and then the recipient cuff was created. The donor and the recipient cuffs were then anastomosed using 4–0 monofilament nonabsorbable suture in an imbricating manner. During anastomosis, carefully assessed the tension on the anastomosis site. Afterward, we proceeded with the left side. Similar to the right side, a left pneumonectomy and implantation were performed using the same methods (Fig. 2). The duration of VA ECMO support was 147 min with 328 min ischemic time. Because of the significant size mismatch, nonanatomic lung volume reduction over the right middle lobe and left upper lobe was necessary. (This is due to recipient and donor size mismatch, not from situs inverse). The patient had no complications post-operation and was discharged on the post-operative day (POD) 12.

### Case 2

Our second patient was a 51-year-old man who had scleroderma-associated interstitial lung disease with situs inversus. His lung condition had deteriorated progressively, leading to a home oxygen therapy requirement in the past year. A bilateral, sequential lung transplant was performed. Similar to case 1, a clamshell incision was made at the fourth intercostal space entry. The patient then received VA ECMO support identical to case 1. The total VA ECMO support time was 155 min with 295 min ischemic time. The patient recovered uneventfully and was discharged on POD 13.

## Discussion

Our case report adds to the existing literature on lung transplantation in situs inversus by providing valuable insight into the surgical management and outcomes of this rare scenario. Prior reports specified the necessity of anastomosing the donor pulmonary artery to the side of the recipient pulmonary artery [6], or performing end-to-end anastomosis by swapping the donor pulmonary artery between the epibronchial and prebronchial locations [3]. A previous study strongly stated the importance of preserving a longer donor bronchus to mitigate any tension [1, 7]. However, in our cases, it was not necessary to apply such surgical modifications. We used the usual surgical techniques including hilum dissection, graft placement, and anastomosis techniques to the reversed anatomy. During the lung transplant procedure, we used VA ECMO to establish a stable, hemodynamic state in both of our patients. Our interpretation of the surgical results emphasizes the feasibility and efficacy of lung transplantation in situs inversus’ patients when the surgical team is equipped with a comprehensive understanding of the anatomical intricacies. By adapting established transplantation procedures to suit the reversed organ positions, we demonstrate that lung transplantation can be a viable treatment option for patients with situs inversus and end-stage lung disease without modifying established transplantation procedures.

## Conclusions

In conclusion, our case report showed that by adapting established transplantation procedures to accommodate the reversed organ positions. Our findings align with previous reports and contribute to the growing body of literature on lung transplantation in situs inversus. The insight gained from our surgical approach provides clinicians, involved in the care of these unique patients, proper guidance for pre-operative planning and intra-operative decision making. By addressing the challenges posed by anatomical variations, we aim to improve outcomes and enhance patient care in this specific patient population.

## Data Availability

The datasets used and/or analyzed during the current study are available from the corresponding author on reasonable request.
